# Assessing the Impact of D‐Dimer on Stroke Diagnosis Within 24 h

**DOI:** 10.1002/jcla.25133

**Published:** 2024-12-05

**Authors:** I‐Shiang Tzeng, Giou‐Teng Yiang, Meng‐Yu Wu, Mao‐Liang Chen

**Affiliations:** ^1^ Department of Research Taipei Tzu Chi Hospital, Buddhist Tzu Chi Medical Foundation New Taipei City Taiwan; ^2^ Department of Emergency Medicine Taipei Tzu Chi Hospital, Buddhist Tzu Chi Medical Foundation New Taipei Taiwan; ^3^ Department of Emergency Medicine, School of Medicine Tzu Chi University Hualien Taiwan; ^4^ Graduate Institute of Injury Prevention and Control Taipei Medical University Taipei Taiwan

**Keywords:** D‐dimer, diagnostic, laboratory, methodology, stroke

Dear Editor,

We read with interest the laboratory analysis and meta‐analysis performed by Ahmad et al. [1]. Using a review of the published literature, the study included controlled/randomized clinical trials (RCTs), retrospective or prospective cohorts, and case‐controlled studies with five or more patients. These studies separated stroke groups from stroke mimic/control groups and reported D‐Dimer values within the 24 h. The analysis revealed a positive effect size for D‐Dimer in the stroke group.

However, we would like to highlight several methodological concerns presented in this paper. First, the estimates of variance among studies may lack precision, especially when a small number of studies are included in the meta‐analysis. This uncertainty was overlooked when applying a conventional normal approximation for random‐effects models, potentially impacting the accuracy of the inferences drawn. The issue of imprecise variances estimates becomes critical when the sample size of included studies is small. Neglecting this uncertainty when integrating the random effects can have detrimental consequences for statistical inferences. To address this concern, the Hartung and Knapp (HK)‐adjusted method should be used to estimate random effects and their confidence intervals (CIs), rather than relying on the standard approach [2, 3]. A previous meta‐analysis compared D‐Dimer levels (ng/ml) between stroke groups and stroke mimics/controls within 6 hours, reporting a standard mean difference (SMD) of 0.49; 95% confidence interval (CI) = [0.29, 0.69]; and *p* < 0.00001 [1]. We reanalyzed the data using random effects models with the HK adjustment. The updated results showed SMD = 0.49; 95% CI = [0.03, 0.95]; and *p* = 0.045 (Figure 1). After the HK adjustment, the *p* value of the overall effect approached the borderline for statistical significance (*p* = 0.05) for D‐Dimer levels in the stroke group compared with the control group. Caution is advised regarding potential small‐study bias when performing meta‐analyses. It is important to note that the 95% CI for the random effect became wider after the HK adjustment, likely due to a decrease in statistical power for the test [4].

**FIGURE 1:**
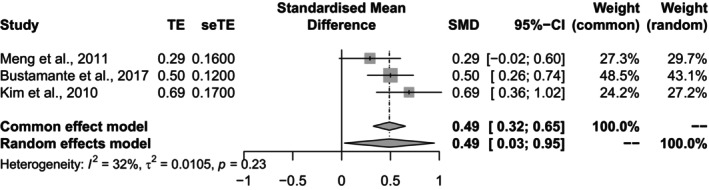
Comparison of D‐Dimer levels (ng/mL) between stroke patients and stroke mimics/controls within 6 h, following the Hartung and Knapp adjustment.

From a clinical perspective, it is essential to recognize that correlation does not imply causation, particularly in nonexperimental studies [5]. When two events, A and B, are related, several possibilities exist: (1) A causes B; (2) B causes A; (3) both A and B have no causal relationship but are influenced by a third factor; or (4) the relationship is coincidental. Confirming true causal relationships between events is a significant challenge and requires empirical evidence to validate hypotheses. Data‐driven analysis can deepen our understanding of disease mechanisms and offer evidence to address clinical challenges. With advanced data‐driven architectures, it is possible to establish strong empirical causality through rigorous analysis of comprehensive data.

RCTs offer the highest level of evidence by providing inferences with strict control of confounding variables [6]. However, even in well‐designed RCTs, certain factors, such as living environments and socioeconomic conditions, cannot be fully controlled. In epidemiological research, no matter how well the study design and measurements are set, the presence of potential and unmeasured confounders cannot be entirely ruled out [7]. This limitation may lead to different outcomes across studies with similar designs and objectives. Additionally, researchers often do not release original data due to privacy concerns.

Fortunately, meta‐analysis, a cutting‐edge data‐driven approach, has been developed to address conflicting research results [8]. By pooling data from multiple studies and accounting for study variance (random effects), meta‐analysis can provide more robust conclusions [8]. Recently, Mendelian randomization (MR) has gained prominence as a method for identifying risk factors and making true causal inferences [9]. MR offers an alternative approach to mitigate the effects of potential and unmeasured confounders in determining disease causality. One of the most common techniques in MR is using two‐stage least squares to adjust for confounders in linear regression models. Figure 2 illustrates the increasing number of instrumental variable (IV) and MR‐related papers published in recent years, demonstrating a growing interest in MR as a tool for understanding disease causality.

**FIGURE 2:**
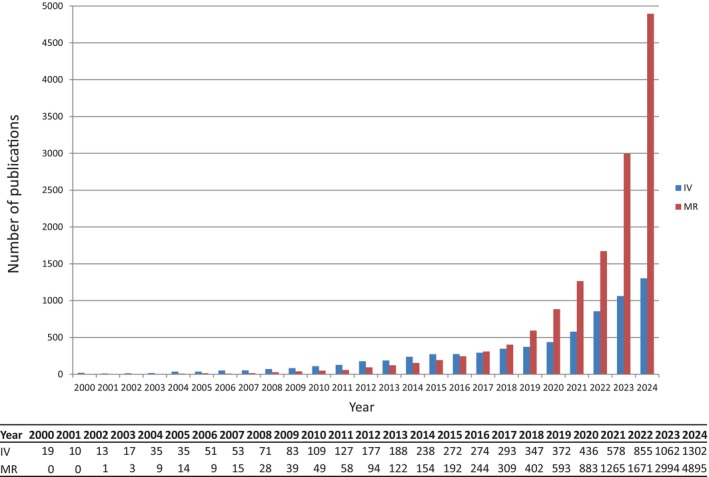
Trends in the use of Mendelian randomization and instrumental variable approaches in the literature over time. PubMed search strategy (October of 2024): For MR analysis, search items included (mendelian random*[tiab]) or (Mendelian Randomization Analysis*[MeSH]); for instrumental variable analysis, search items included (instrumental variable*[tiab]).

There are some limitations that need to be addressed in the study. The authors reported that stroke patients had higher D‐Dimer values on presentation than stroke mimics/controls, based on their meta‐analysis. However, it is important to note that the subgroup analysis included a small number of studies (*n* = 3; Figure 1) [1], which increases the likelihood of bias due to the limited sample size. While the results remained similar after adjustment (SMD = 0.49), the *p* value increased (*p* = 0.045), reflecting the borderline statistical significance. It is crucial to remember that the study size should ideally include more than five studies (> 5) to ensure robust results [2, 3]. In this case, the HK adjustment was applied to weighted least squares regression models. Another significant limitation is that correlation does not imply causation [5]. The authors could consider employing genome‐wide association studies using the MR approach to investigate the causal relationship between D‐Dimer levels and stroke diagnosis or prognosis in future research [10]. In summary, while this study provides valuable insights into the association between D‐Dimer levels and stroke diagnosis, it highlights the need for more extensive research and rigorous methodologies to refine the mean difference of D‐Dimer values as a diagnostic tool, either alone or in conjunction with other interventions.

## Conflicts of Interest

The authors declare no conflicts of interest.

## Data Availability

The data supporting the findings of this study are included within the article.
